# A Guide to Non-Alcoholic Fatty Liver Disease in Childhood and Adolescence

**DOI:** 10.3390/ijms17060947

**Published:** 2016-06-15

**Authors:** Jonathan L. Temple, Paul Cordero, Jiawei Li, Vi Nguyen, Jude A. Oben

**Affiliations:** 1 Faculty of Life Sciences and Medicine, King’s College London, Strand, London WC2R 2LS, UK; jonathan.temple@kcl.ac.uk; 2 Institute for Liver and Digestive Health, University College London, Rowland Hill Street, London NW3 2PF, UK; jiawei.li.10@ucl.ac.uk (J.L.); v.nguyen@ucl.ac.uk (V.N.); 3 Department of Gastroenterology and Hepatology, Guy’s and St Thomas’ Hospital, NHS Foundation Trust, Westminster Bridge Rd., London SE1 7EH, UK

**Keywords:** NAFLD, steatosis, obesity, children, adolescent

## Abstract

Non-Alcoholic Fatty Liver Disease (NAFLD) is now the most prevalent form of chronic liver disease, affecting 10%–20% of the general paediatric population. Within the next 10 years it is expected to become the leading cause of liver pathology, liver failure and indication for liver transplantation in childhood and adolescence in the Western world. While our understanding of the pathophysiological mechanisms underlying this disease remains limited, it is thought to be the hepatic manifestation of more widespread metabolic dysfunction and is strongly associated with a number of metabolic risk factors, including insulin resistance, dyslipidaemia, cardiovascular disease and, most significantly, obesity. Despite this, ”paediatric” NAFLD remains under-studied, under-recognised and, potentially, undermanaged. This article will explore and evaluate our current understanding of NAFLD in childhood and adolescence and how it differs from adult NAFLD, in terms of its epidemiology, pathophysiology, natural history, diagnosis and clinical management. Given the current absence of definitive radiological and histopathological diagnostic tests, maintenance of a high clinical suspicion by all members of the multidisciplinary team in primary and specialist care settings remains the most potent of diagnostic tools, enabling early diagnosis and appropriate therapeutic intervention.

## 1. Introduction

Non-Alcoholic Fatty Liver Disease (NAFLD) encompasses a spectrum of chronic liver disease, characterised by excessive hepatic fat accumulation (steatosis) in the absence of significant alcohol consumption, occurring with or without hepatic inflammation and fibrosis [1]. Simple or bland hepatic steatosis describes the abnormal accumulation of fat in >5% of hepatocytes, without evidence of hepatocellular injury or fibrosis. A significant proportion of patients with hepatic steatosis, however, progress to a more advanced form of the disease, Non-Alcoholic Steatohepatitis (NASH), where steatosis coexists with hepatocellular injury and inflammation, which can precipitate hepatic necrosis, fibrosis and cirrhosis, as well as a significantly increased risk of hepatocellular carcinoma [1,2,3].

NAFLD is thought to be a hepatic manifestation of more widespread and underlying metabolic dysfunction and is strongly associated with a number of metabolic risk factors, including insulin resistance, dyslipidaemia, cardiovascular disease and, most significantly, obesity [2,4,5]. Our understanding of the pathophysiological mechanisms underpinning these relationships, however, remains incomplete.

While detailed clinico-pathological descriptions of NAFLD in adults can be found in the literature as far back as the 1850s, the first case of paediatric NAFLD was reported in 1983 by Moran *et al.* [6,7]. It is now the most prevalent form of chronic liver disease in childhood and adolescence, affecting approximately 10%–20% of the general paediatric population. Within the next 10 years, paediatric NAFLD is expected to become the most prevalent cause of liver pathology, liver failure and indication for liver transplantation in childhood and adolescence in the Western world [8,9,10,11,12,13].

Despite this, ”paediatric” NAFLD remains under-studied, under-recognised and, potentially, undermanaged [14]. Important gaps remain in our overall approach to screening, diagnosis, management and follow-up, particularly during the transition between paediatric and adult clinical services [15]. More accurate epidemiological and pathophysiological data derived from larger longitudinal cohort studies are needed in order to better determine the true prevalence and natural history of paediatric NAFLD among different ethnic groups, aiding the selection and widespread implementation of more effective therapeutic interventions [13,16]. Recognition, first, of the occurrence of NAFLD in the paediatric population and, second, the differences in its clinical presentation, pathophysiology, histology and prognosis when compared to adult disease, is of critical importance.

## 2. Clinical Presentation of Paediatric Non-Alcoholic Fatty Liver Disease (NAFLD)

Although cases of paediatric NAFLD and NASH-related cirrhosis have been reported in patients as young as 2 and 8 years old, respectively, most usually present clinically above the age of 10 years. The mean age of diagnosis is 11–13 years old [11,12,17]. However, NAFLD often remains asymptomatic until significant damage to the liver and/or other systems has occurred or coincident acute liver injury manifests worse clinical outcomes than would otherwise be expected or NAFLD-associated comorbidities, including insulin resistance and Type II Diabetes Mellitus, develop. Diagnosis, therefore, is often incidental on physical examination or routine blood testing, accounting for approximately 7%–11% of abnormal liver function tests (LFTs) and 74% of liver biopsies in obese patients with metabolic risk factors [8,9].

Children may also report non-specific symptoms, including abdominal pain due to stretching of the liver capsule, fatigue, irritability, headaches and difficulty concentrating [12,14]. Hepatomegaly may be appreciated on manual palpation in up to 50% of cases but can be difficult to discern in obese patients. Acanthosis nigricans, a clinical marker of hyperinsulinemia that can manifest on the back of the neck, intertriginous areas or joints, has been reported in 33%–50% of children with biopsy-proven NAFLD [8,9,11,17,18].

A landmark study of 742 autopsy specimens from children in San Diego County (CA, USA) between 1993 and 2003 found evidence of NAFLD in 17.3% of children aged 15–19 years old [9]. This is consistent with other more recent studies [11,19,20], including one involving 995 adolescents aged 17 years old, which reported a prevalence of NAFLD of greater than 15% [21]. The true prevalence of paediatric NAFLD, however, is difficult to determine and may be even higher, given the marked variations in the populations studied, in terms of age, ethnicity, the diagnostic parameters applied and clinical bias with regards to the ”appropriateness” of diagnosing NAFLD in children, as well as the general paucity of research.

Certainly, the prevalence of NAFLD in childhood and adolescence has greatly increased in recent decades, in the wake of rising levels of childhood obesity [22]. Paediatric NAFLD is strongly associated with a number of metabolic risk factors, including increased insulin resistance, dyslipidaemia, cardiovascular disease and, most significantly, visceral adiposity [12,22,23,24]. A number of studies now suggest the prevalence of NAFLD in overweight and obese youth to be up to 70%, compared to 7% in those of normal weight [25,26]. Severe obesity (>95th centile for age and gender-adjusted body mass index) is also associated with more adverse clinical outcomes and greater risk of progression to NASH and cirrhosis in childhood [14].

Below 3 years of age, obesity does not usually produce hepatic steatosis and, as such, its incidence may well indicate more severe underlying metabolic dysfunction with worse prognosis [17]. Therefore, ‘brightness’ of the liver on ultrasound or increased aminotransferases in this age group requires a detailed clinical workup, to exclude many rare metabolic or systemic diseases that may also present with hepatic steatosis, collectively referred to by some authors as the ”NASH trash bin” [17].

While simple steatosis carries a minimal risk of cirrhosis and liver failure in adults, it appears to follow a more aggressive course in paediatric cases, with many children progressing to NASH and hepatic fibrosis either in childhood or early adulthood [27,28]. Paediatric patients with more advanced fibrosis on liver biopsy tend to have more hepatic complications and a worse prognosis, particularly regarding the risk of cirrhosis [29]. A high clinical suspicion should therefore be maintained, particularly in children more than 10 years old who are overweight or obese and have a waist circumference above the 95th centile, in the context of other metabolic risk factors, abnormal LFTs and a family history of severe NAFLD [17].

Some studies have suggested, however, that normal-weight individuals with NAFLD appear to present at a younger age than those who are overweight or obese and demonstrate a decreased association with components of the metabolic syndrome, such as hypertension and insulin resistance [30,31]. This has given rise to the controversial hypothesis that paediatric NAFLD might, in fact, represent a group of related but pathophysiologically distinct clinical phenomenologies.

### 2.1. NAFLD and Obesity

The single greatest risk factor for paediatric NAFLD is obesity, with an estimated prevalence in overweight and obese youth of 50%–80% compared to 2%–7% in children of normal weight [25,26]. A recent cross-sectional study of 182 obese sedentary children and adolescents demonstrated a positive correlation between increased abdominal fat and the incidence of NAFLD, independently of insulin resistance and dyslipidaemia [32]. Central obesity has also been shown to reliably predict evidence of NAFLD on ultrasound and aminotransferase elevation in a cohort of more than 11,000 obese patients aged 6–18 years old [33]. A further study by Manco *et al.* [34] reported that 92% of paediatric NAFLD patients had a Body Mass Index (BMI) higher than the 85th centile and 84% had a waist circumference greater than the 90th centile. Moreover, significant correlation between waist circumference, total fat mass and intra-abdominal adipose tissue and the incidence of NAFLD was also reported in a cross-sectional study of 145 patients aged 11–17 years [10]. Waist circumference may, therefore, represent an interesting and reliable screening tool in paediatric NAFLD.

While obesity is thought to cause an overabundance of circulating free fatty acids, increasing hepatic steatosis, as well as contributing to the development of insulin resistance, the exact pathophysiological mechanisms by which obesity increases the risk of paediatric NAFLD remain poorly understood [14,35]. Indeed, not all children who are obese develop NAFLD, suggesting that other factors may inform risk such as the preferential deposition of visceral, as opposed to subcutaneous, adipose tissue [6,36].

Visceral adipose tissue is the primary source of hepatic fat in adults, contributing 59% of the triglyceride found in the liver; the main component of fat accumulation in NAFLD [9]. Increasing evidence also suggests that adipose tissue fulfils important and distinct endocrine functions, producing multiple pro-inflammatory adipocytokines, including TNF-α, IL-6, leptin and adiponectin, which are implicated in the clinical manifestation of NAFLD and its progression to NASH and cirrhosis [37,38]. Pentoxifylline, a phosphodiesterase inhibitor and non-specific TNF-α pathway antagonist, has been shown to promote a reduction in serum Alanine Aminotransferase (ALT) levels and improvement of the histological features of NASH in adult patients [12,39]. Other TNF-α inhibitors, such as infliximab, a selective chimeric monoclonal antibody against TNF-α, and resveratrol, a polyphenol with anti-inflammatory activity, have shown interesting results in adult clinical trials [12,39].

**Figure 1:**
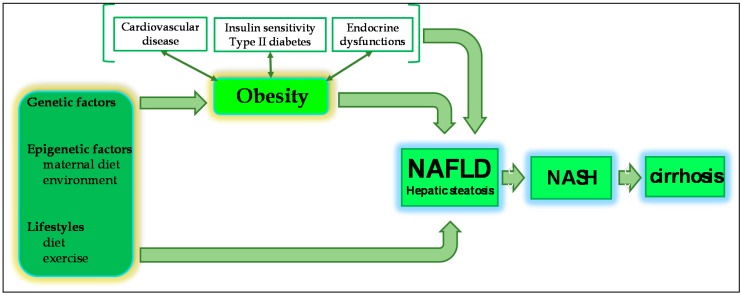
Obesity and Non-Alcoholic Fatty Liver Disease (NAFLD). Abbreviations: NAFLD: non-alcoholic fatty liver disease; NASH: non-alcoholic steatohepatitis.

Furthermore, abdominal visceral adipose tissue has peculiarities of its own, including higher lipolysis and greater release of adipokines [32]. There is also evidence to suggest that, as the adipose bed expands, adipocytes suffer from a micro-hypoxic environment, due to insufficiency of its vascular network, resulting in cell injury and death and consequent upregulation of the pro-inflammatory cascade [9]. Circulating adipokines also appear to promote specific patterns of lipid storage and metabolic stress, which in turn activate signalling cascades that induce oxidative stress and trigger a local and/or systemic inflammatory response [35]. However, visceral adipose mass is much less developed in children, compared with adults, though it accumulates rapidly with weight gain, particularly in males. It has, therefore, been suggested that subcutaneous adipose tissue, although less metabolically active than visceral adipose tissue, may play a greater role in paediatric NAFLD [6,36]. Indeed, recent reports describe specific differences in the distribution of subcutaneous adipose tissue between adolescents with NAFLD and those without. These differences are apparent from three years old but not at birth, suggesting that the first three years of life might represent a critical window in which various interactions between genetic, environmental, epigenetic and metabolic factors contribute to the future risk of NAFLD [6] (Figure 1).

### 2.2. Hepatic Complications of NAFLD

Non-Alcoholic Steatohepatitis (NASH) is commonly considered a more advanced form of NAFLD, where steatosis coexists with hepatocellular injury and inflammation, precipitating hepatic necrosis, fibrosis and cirrhosis and a significantly increased risk of hepatocellular carcinoma [1,19]. NASH significantly increases both overall and liver-related mortality, with the most common causes of death being cirrhosis and liver failure, neoplasia, sepsis, variceal haemorrhage and cardiovascular disease [11]. Long-term follow-up studies have shown that, in adults, NASH increases overall mortality by 35%–58% compared with age and sex-matched controls, while liver-related mortality is increased 9–10 fold [40,41,42]. NAFLD is, by far, the most common cause of hepatic fibrosis and cirrhosis in adults and children with unexplained or cryptogenic increases in serum alanine aminotransferase. However, advanced fibrosis can readily coexist with normal serum aminotransferase levels and has been reported in up to a third of patients with isolated simple steatosis [11].

#### 2.2.1. Fibrosis

Approximately 25% of paediatric patients will progress to NASH, though the risk increases significantly in the context of obesity [43]. For example, a recent study of 24 severely obese bariatric adolescent patients found 63% had definitive NASH and a further 25% had ”borderline” NASH [44]. Hepatic fibrosis has been documented retrospectively in more than one third of adult patients with NASH [11]. In a national multi-centre study, advanced fibrosis was reported at the time of diagnostic liver biopsy in nearly one in seven children with NAFLD [43]. Another study reported similar findings, with 17% of children with NAFLD having advanced fibrosis. After adjusting for fibrotic confounders, NASH appears to have a fibrotic potential similar to that of chronic Hepatitis C [11,45]. The main predictors of the severity of fibrosis are increasing age, BMI > 28–30 kg/m^2^, hypertension, the degree of insulin resistance and diabetes [11]. Hepatic fibrosis also appears more prevalent in adolescents with severe obesity (83% *vs.* 29% in adults), further suggesting that paediatric patients, especially those who are obese, tend to follow a more aggressive clinical course than adults with NAFLD [14].

#### 2.2.2. Cirrhosis

After 10 years, the risk of cirrhosis in adult patients with NASH is 15%–25%. Once cirrhosis is established, 30%–40% of these die within another 10 years [23]. Current evidence suggests that children have similar risks of progressing from NASH to decompensated end-stage liver disease, requiring transplantation [44].

End-stage NASH is a frequent and important cause of cryptogenic cirrhosis, mainly because hepatic fat accumulation and evidence of hepatocellular injury can disappear at this advanced stage; a phenomenon sometimes referred to as ”burned out” NASH [11]. It has been shown that if a diagnosis of NASH were made on the basis of past or present exposure to metabolic risk factors, such as obesity, diabetes and hypertension, when histological signs are lacking, approximately 30%–75% of cryptogenic cirrhosis could be attributed to ”burned-out” NASH. Liver failure is often the first presentation of patients with cirrhotic NASH and usually occurs after 7–10 years in adults but due to its quicker development, it may occur even more rapidly in paediatric cases [11].

#### 2.2.3. Hepatocellular Carcinoma

Hepatocellular carcinoma can occur in both cirrhotic and, it appears, non-cirrhotic NASH. Its prevalence is greater still in obese or diabetic NAFLD patients [46,47]. In a study cohort of 285,884 boys and girls in Copenhagen who were followed for over three decades, higher body mass index (BMI) in childhood was associated with an increased risk of primary liver cancer in adulthood [48]. The hazard ratio (95% CI) of adult liver cancer was 1.20 (1.07–1.33) and 1.30 (1.16–1.46) per unit BMI *z*-score at 7 and 13 years, respectively. Similar associations were found for boys and girls for hepatocellular carcinoma only, across years of birth, and after accounting for diagnoses of viral hepatitis, alcohol-related disorders and biliary cirrhosis [48]. There is also, likely, a chronic underestimation of the proportion of NASH progressing towards end-stage liver disease, as many patients are no longer listed because of the co-occurrence of associated diseases, including obesity, cardiovascular disease and diabetes [11].

### 2.3. Extra-Hepatic Complications of NAFLD

While NAFLD is not a formal component of the diagnostic criteria for the metabolic syndrome, they do share common major risk factors, including central obesity, high serum triglycerides and high-density lipoprotein cholesterol (HDL-C), hypertension and insulin resistance, as well as altered glucose and lipid metabolism. Nearly 90% of NAFLD patients have at least one feature of the metabolic syndrome and up to 33% meet the complete diagnosis [4,18,49].

What is clear is that patient outcomes worsen when both conditions co-occur in an apparently synergistic manner [4,24,35,44] The presence of the metabolic syndrome, also, is a strong clinical predictor of NASH, particularly in overweight and obese paediatric patients [19,32]. This has led some to describe paediatric NAFLD in terms of either the hepatic manifestation or precursor of the metabolic syndrome [12,44,49]. Others, however, have suggested that both conditions may feed into one another, creating a vicious cycle of worsening metabolic disease, likely indicative of more widespread underlying metabolic dysfunction [35]. However, while we might infer that there exists significant overlap between the pathophysiological mechanisms that underlie these two conditions, their nature and extent remain poorly understood [4,18,49].

#### 2.3.1. Cardiovascular Disease

NAFLD is an independent risk factor for coronary artery disease, as well as being strongly associated with a number of other cardiovascular risk factors, including multi-organ insulin resistance, dyslipidaemia and impaired flow-mediated vasodilatation [50]. Significant carotid atherosclerosis has been shown to occur 5–10 years earlier in patients with NAFLD than in those without and, in cases of biopsy proven NAFLD, hepatic steatosis is associated with increased carotid artery intima-media thickness and the presence of carotid plaques [11]. Biochemical surrogates of NAFLD, γ-glutamyl transferase (GGT) and ALT, predict the incidence of coronary artery disease and other cardiovascular disease, which is further elevated in NAFLD patients who suffer co-morbidly with diabetes mellitus [11]. Furthermore, in adults, NAFLD has been associated with myocardial insulin resistance, altered cardiac energy metabolism, abnormal left ventricular structure and impaired diastolic function; the duration and severity of these abnormalities in cardiac function likely contributing to the increased risk of heart failure and cardiovascular mortality in obese patients and, particularly, those with NAFLD [50]. Indeed, adult patients with NAFLD are at a significantly higher risk of cardiovascular mortality than the general population, with cardiovascular disease being the most common cause of death in NAFLD patients [11,44].

Cardiac functional abnormalities have also been reported in obese adolescents that were independent of traditional cardiac risk factors (*i.e.*, high systolic and diastolic pressures, total and low-density lipoprotein cholesterol and BMI) and correlated with insulin resistance [50]. One study assessing 50 children with biopsy-proven NAFLD using 24 h blood pressure monitoring and Doppler echocardiography parameters reported instances of cardiac dysfunction that were detectable in early NAFLD and were linked to no other cardiovascular or metabolic alteration other than liver damage. Left ventricular hypertrophy was present in 35% of patients, concentric remodelling in 14% and left atrial dilatation in 16%. Furthermore, children with simple steatosis showed lesser cardiac alterations than NASH patients [51]. Pacifico *et al.* [52] went on to demonstrate that even asymptomatic obese children with NAFLD exhibit early left ventricular diastolic and systolic dysfunctions, becoming more severe in patients with NASH. Hence, as NAFLD advances, the extent of cardiovascular dysfunction increases, with several other studies demonstrating greater endothelial dysfunction, an early proatherogenic lesion, and carotid intima thickness in NASH than in simple steatosis.

Elsewhere, Nobili *et al.* [53] have demonstrated that the severity of liver injury is strongly associated with the presence of a more atherogenic lipid profile, in terms of triglyceride/high density lipoprotein cholesterol (HDL), total cholesterol/HDL and low density lipoprotein (LDL)/HDL ratios. A further study of 548 children with a high triglycerides/HDL ratio reported an increased risk of insulin resistance that correlated independently, with more advanced NAFLD [54].

#### 2.3.2. Insulin Resistance and Type II Diabetes Mellitus

Insulin Resistance (IR) is the most common metabolic abnormality associated with NAFLD and, perhaps, the most useful indicator of disease severity and progression in adults and children [19,49]. The severity of IR is strongly associated with the amount of hepatic fat accumulation, independently of global and intra-abdominal adiposity and the prevalence of NAFLD is greater in patients with hyperglycaemia and type II diabetes, with evidence of NAFLD present on ultrasound in up to 70% of clinical cases [14,32].

The key question remains, however, as to whether this relationship is causal or whether hepatic fat accumulation is, itself, a consequence of insulin resistance. On the one hand, hepatic steatosis and impairment reduces insulin clearance and, over time, greater insulin resistance [11]. Indeed, in NAFLD, steatosis and hepatic IR have been shown to occur in advance of peripheral IR, suggesting that the former is the primary defect in the development of the latter. Hepatic steatosis has, in turn, been shown to exacerbate insulin resistance by interfering with the phosphorylation of insulin receptor substrates, with the amount of hepatic steatosis correlating with the severity of IR [11,12].

On the other hand, insulin is an anabolic hormone that promotes glucose uptake in the liver, skeletal muscle and adipose tissue [9,10,12]. Increasing insulin resistance precipitates a reduction in glucose uptake by the liver and a compensatory increase in circulating levels of insulin. This drives increased hepatic and peripheral glycogenesis and lipogenesis, via sterol regulatory binding element (SREBP-1c) mediated upregulation of several prolipogenic genes, as well as impairing hepatocytic fatty acid metabolism [9,10,12]. As a result, circulating free fatty acids become increasingly abundant, most being taken up by the liver, where they are invariably processed into triglycerides and deposited within the cytoplasm of hepatocytes in large triglyceride-filled vacuoles, manifesting hepatic steatosis. As insulin resistance develops, high serum glucose levels also activate the carbohydrate responsive element binding protein, which further promotes lipogenesis and hepatic fat deposition [9].

It has also been suggested, therefore, that insulin resistance and hyperglycaemia may induce fibrosis directly or via upregulation of connective tissue growth factor, the generation of advanced glycation end products or through upregulation of pro-inflammatory cytokine production [11,55].

Controversially, others have sought to describe hepatic steatosis in terms of an adaptive, albeit imperfect, hepatic response to hepatic stress that forestalls the onset of NASH, albeit one that, in children, appears less effective and more prone to its own complications [9,11,56]. Indeed, Choi and Diehl suggested that the formation of lipid droplets may actually be protective by sequestering toxic free fatty acids in the form of triglycerides but, that when this buffer exceeds its capacity, certain free fatty acids begin to exert their toxic effect [57]. Work done in mice demonstrated that when triglyceride synthesis was inhibited, hepatic fat accumulation decreased but liver damage worsened, as measured by necroinflammation and fibrosis [58]. Conversely, up-regulation of diacylglycerol O-acyltransferase 2 (DGAT2) resulted in increased hepatic steatosis and was associated with a significant increase in liver inflammatory markers. Free fatty acids and their lipotoxic intermediates have been implicated in the promotion of inflammation, endoplasmic reticular stress, mitochondrial dysfunction and oxidant stress. These processes are injurious to hepatocytes, which, in turn, release pro-inflammatory cytokines and reactive oxygen species as they die, driving further hepatic inflammation [9]. Therefore, we are forced to consider whether steatosis, while a useful biomarker of ongoing injurious and fibrotic mechanisms resulting in disease progression, should be considered at all a therapeutic target and whether such interventions are in actual fact more damaging [11]. Instead, Wanless and Shiota [59] postulated that extracellular fat accumulation after hepatocyte necrosis might also impair hepatic blood flow through hepatic veins but this remains unproven.

#### 2.3.3. Other Endocrine Disorders

There is evidence to suggest that other endocrine disorders, such as hypothyroidism, hypogonadism, hypopituitarism and polycystic ovary syndrome, independently of obesity, are important risk factors for NAFLD [11,60,61]. Several studies have addressed the association between thyroid dysfunction and NAFLD. Pacifico *et al.* [62] were the first to provide evidence of such a link between NAFLD, thyroid function and the metabolic syndrome in childhood, demonstrating a positive correlation between thyroid function tests, thyroid stimulating hormone (TSH) in particular, and the incidence of NAFLD in overweight and obese children, independently of visceral adiposity. Subsequently, Torun *et al.* [61] showed that TSH levels significantly increase in accordance with the extent of steatosis on ultrasound and ALT and BMI.

## 3. The Pathogenesis of NAFLD

Traditionally, the pathogenesis of NAFLD has been described in terms of a two-hit hypothesis, where hepatic steatosis sensitises the liver to the effects of oxidative stress and the action of various pro-inflammatory cytokines, which would, over time, drive the development of necroinflammation, fibrosis and, ultimately, cirrhosis [11,12]. However, increasing evidence of the complexity and inter-relatedness of numerous pathophysiological mechanisms, both hepatic and extra-hepatic, implicated in the development and progression of NAFLD, has precipitated a change in thinking. The now widely accepted “multiple-hit model” instead approaches NAFLD in terms of a hepatic manifestation of more widespread metabolic dysfunction, brought about through the interaction of numerous genetic and environmental factors, as well as changes in cross-talk between different organs, including adipose tissue, the pancreas, gut and liver [4,6,12,44]. Obesity and insulin resistance have repeatedly been suggested as the first “true” hits.

The development of NAFLD in children, in particular, it seems is characterised by an intricate network of interactions between resident hepatic and recruited cells, such as Kupffer cells, T cells and hepatic stellate cells, which drive disease progression alongside other infiltrating inflammatory cell-derived factors released either as a direct result of hepatic steatosis, hepatocyte injury and apoptosis or as an indirect response to hepatic damage and/or gut-derived bacterial products acting on Toll-like pattern recognition (TLR) receptors [63,64]. Indeed, dysregulation of pro-inflammatory cytokines and adipokines are almost universally detected in NAFLD patients, while endoplasmic reticular, mitochondrial and cytokine-mediated oxidative stress and hepatocytic apoptosis appear to contribute to the development of NASH [65,66,67]. TLR antagonists may also, in time, prove effective therapeutic agents for NASH; a potential that mandates further study [12].

Hepatic Stellate cells are considered the main extracellular matrix-producing cells during NASH development and are activated following hepatocyte injury and apoptosis, mediating the development of hepatic fibrosis and, if activation is chronic, cirrhosis. Hepatic Progenitor Cells (HPC), the resident stem cell population within the liver, have recently been shown to be expanded in paediatric NAFLD [66]. They appear to play a role in the liver’s response to oxidative stress, their levels correlating with fibrosis and NASH progression [66]. Furthermore, HPCs can undergo an epithelial-mesenchymal transition, resulting in a profibrogenic myofibroblast-like cell population, a process involving the Hedgehog signalling pathway [68].

Kupffer Cells are important regulators of the biological exchanges between hepatocytes and other liver cells, engaging and sustaining the action of neutrophils, natural killer T lymphocytes (NKT) and blood monocyte-derived macrophages, as well as phagocytosing and removing microorganisms, apoptotic cells and cell debris themselves, processing and presenting antigens to attract cytotoxic and regulatory T cells, contributing to adaptive immunity. Increasing evidence suggests that they fulfil many diverse roles in the pathogenesis and progression of NAFLD, including the regulation of immune tolerance and lipid homeostasis [63,69]. Indeed, Stienstra *et al.* [70] further demonstrated the integral role of Kupffer cells in regulating hepatic triglyceride storage and the promotion of hepatic steatosis via IL-1β-mediated suppression of perioxisome proliferator-activated receptor-α (PPAR-α) activity, while others have reported that Kupffer cell depletion, in a murine experimental model of NASH, prevented hepatic fat accumulation and liver damage [63].

Several studies have described subsequent changes in the frequency and/or functionality of peripheral T cell subpopulations, manifesting an altered phenotype of infiltrating and circulating immune cells that appears to be distinct between adult and paediatric NASH [64]. Several studies have reported a predominance of CD8+ T cells over CD4+ and CD20+ subpopulations undergoing activation in paediatric NASH, in association with increased levels of IFN-γ within the hepatic microenvironment, a high number of infiltrating neutrophils in correlation with Reactive Oxygen Species (ROS) generation in peripheral neutrophils and further alterations in the phenotype and functionality of circulating lymphocytes and neutrophils compared with age-matched controls. By contrast, CD8+ cells were a minor component of Natural Killer (NK) and NKT cells in adult NASH [19,64]. The molecular and immunological phenomenology of these systems both locally and systematically, in both paediatric and adult NASH, are complex and are only just beginning to be recognised, let alone understood.

Increasing evidence suggests that dysregulation of the autonomic nervous system innervation of the liver fulfils a critical role in the progression of simple steatosis to NASH and cirrhosis. Indeed, Hepatic Stellate Cell (HSC) autonomic receptors are reportedly upregulated in the livers of adult NAFLD patients and may represent another potential target for future anti-fibrotic therapies [71,72].

### 3.1. Genetics of Paediatric NAFLD

Over the last decade, with the advent of next-generation sequencing technologies, polymorphisms associated with the incidence and severity of paediatric NAFLD have been identified in numerous genes involved in lipid metabolism, insulin sensitivity, oxidative stress, regulation of the immune system and the development of fibrosis [4,73]. Furthermore, evidence of the strong genetic contribution to the pathogenesis of paediatric NAFLD comes from reports familial clustering of metabolic risk factors, including obesity, insulin resistance and type II diabetes. One study of children with biopsy-proven NAFLD, for example, reported that 59% of their siblings and 78% of their parents were found to have evidence of hepatic steatosis on MRI, significantly more than in relatives of age and BMI-matched children without NAFLD [74].

The prevalence and genetic variants associated with NAFLD also vary between different ethnic groups, likely affecting the heritability of metabolic risk factors that contribute to individual susceptibility to the disease [75]. Hispanic children demonstrate the highest prevalence of NAFLD (36%), greater than that of Afro-Caribbeans (14%), Asians (10.2%) and non-Hispanic whites (8.6%) despite these populations exhibiting similar obesity rates [13]. Hispanic patients have also been shown to be at higher risk of type II diabetes and tend to display more features of the metabolic syndrome than non-Hispanic whites, which may further contribute to their greater risk. It has also been suggested that differences in body fat distribution among Afro-Caribbean children, who notably have more subcutaneous fat and less visceral fat and consequently a lesser predisposition towards hepatic fat accumulation, may explain their lower prevalence of NAFLD. Indeed, visceral adiposity is less associated with NAFLD among Afro-Caribbean adolescents than among non-Hispanic whites. Furthermore, insulin resistance appears less tightly linked to visceral adiposity in Afro-Caribbean children with NAFLD and tends to be more associated with the extent and severity of liver damage. Conversely, the extent to which the relationship between insulin resistance and NAFLD severity varies between Hispanics and non-Hispanic whites appears negligible [13,75].

A recent genome-wide association study (GWAS) conducted by the Genetics of Obesity-Related Liver Disease Consortium identified robust associations between polymorphisms of the genes neurocan (NCAN), lysophospholipase-like 1 (LYPLAL1), glucokinase regulatory protein (GCKR) and protein phosphatase 1 regulatory subunit 3b (PPP1R3B) and NAFLD in adults of European ancestry [76]. However, Palmer *et al.* [16] reported that the allele frequency and effect size of PNPLA3 rs738409, NCAN rs2228603, LYPLAL1 rs12137855, GCKR rs780094 and PPP1R3B rs4240624 varied between adult patients of African and Hispanic ethnicity. Hernaez *et al.* [77] also reported a lack of consistency of these variants in the NHANES III study population of multiple ethnicities. Another GWAS conducted by Romeo *et al.* [78] also found that the PNPLA3 rs738409 variant was seen more commonly in Hispanics than in other ethnic groups and was associated with increased liver fat and hepatic inflammation, whereas PNPLA3 rs6006460 was seen more commonly in Afro-Caribbeans and correlated with lesser hepatic fat accumulation. This has been confirmed by another study of 83 obese children using MRI to quantify hepatic lipid content [79]. Further studies have also shown PNPLA3 rs738409 to be associated with greater hepatic steatosis and disease severity, as well as earlier clinical presentation [55,80].

The fat mass and obesity associated (FTO) gene variant rs9939609 has also been associated with increased risk of NAFLD and the Melanocortin 4 Receptor (MC4R) rs12970134 variant with increased ALT levels, independently of BMI, in children aged 7–18 years old with NAFLD [81]. Other genetic variants associated with NASH, hepatic fibrosis and the severity of liver damage in both adults and children have been described in genes involved in lipid metabolism, such as adiponutrin/patatin-like phospholipase domain-containing 3 (PNPLA3), Lipin 1 (LPIN1), adipoprotein C3 (APOC3), endocannabinoid receptor CB2, as well as the hereditary hemochromatosis (HFE) gene [55,82]. For example, PNPLA3 rs738409 has been associated with the presence and severity of hepatic steatosis in numerous studies, independently of insulin resistance or inflammatory changes, lobular inflammation and perivenular fibrosis in both adult and paediatric NAFLD [55,77,80,83]. Other genes associated with progression to NASH relate to oxidative stress and include the rs4880 variant of manganese-dependent superoxide dismutase (SOD2) gene, the rs1801278 variant of insulin receptor substrate-1 (IRS-1) and the rs3750861 variant of tumour suppressor gene Kruppel-like factor 6 (KLF-6) [55].

Our understanding of the mechanisms by which variation in these genes affects the incidence and progression of NAFLD, however, remains limited. PNPLA3, for example, is most robustly expressed in the liver. Its expression appears to be directly related to nutritional intake, being down-regulated in the fasting state and upregulated during feeding. *In vitro* and mouse models have shown that SREBP-1, which is activated by insulin, induces PNPLA3, which then promotes lipogenesis and modulates glucose homeostasis [84]. Additionally, cytochrome P450 oxidative enzyme family 2 subfamily E member 1 (CYP2E1) is a risk factor for oxidative stress and may be implicated in NAFLD [85,86]. Polymorphism of the cytokine Interleukin 6 (IL-6) have been associated with serum of liver damage markers [87]. Variants in the UGT1A1 gene (Gilbert syndrome) have also been shown to contribute to increased bilirubin levels, thus reducing the risk for NAFLD onset and development [88].

Accumulating evidence also suggests the involvement of the endocannabinoid system in NAFLD, which has many diverse roles in humans. For example, in studies of obese children with steatosis and biopsy-proven NAFLD, a functional variant of the otherwise hepatoprotective cannabinoid receptor 2 (CB2), Q63R, was associated with elevated serum aminotransferase levels [89]. Others have suggested that the CB2 Q63R variant fulfils a critical role in modulating hepatic inflammation in obese children, manifesting an increased susceptibility to liver damage in these patients [82].

Given that the effect of genetic variants tends to be more pronounced in children than in adults, due to a lack of confounding long-term environmental exposures, the investigation of relevant genetic variants associated with paediatric NAFLD, whilst not, at present, consequent to our clinical approach, may prove instructive for both paediatric and adult disease as our understanding of their pathophysiological role increases.

### 3.2. Maternal Diet, Intrauterine Growth and Neonatal Diet

In recent years, the critical role of maternal physiology and metabolism during the perinatal, foetal and even pre-conceptual phases of development in predisposing the unborn towards developing NAFLD within their own lifetimes and making it more likely that they will progress to NASH, has become ever more apparent [69,90,91,92].

This phenomenon, referred to as developmental programming, appears to be driven by the complex interaction of diverse communities of epigenetic modifications at key genes, which change the phenotypic characteristics of different cell types, hence the offspring’s metabolic profile [93]. Recent evidence even suggests that, in addition to the effects of epigenetic programming upon first generation offspring, subsequent generations may also be affected [94].

A greater understanding of the molecular phenomenology underlying maternal epigenetic programming in obesity may well lead to the development of effective therapeutic interventions that may be targeted during key developmental windows to ameliorate the risk of maternal obesity and maternal diet to the unborn. Several studies have now demonstrated that controlled maternal weight loss prior to pregnancy is effective in reducing their offspring’s lifetime risk of developing NAFLD, which is of particular relevance in the context of the rising global prevalence of obesity among women of childbearing age [94]. However, specific and coherent guidelines regarding when and how to effectively intervene in clinical practice have yet to be defined.

Several studies have also found an association between intrauterine growth restriction (IUGR) and obesity, dyslipidaemia, hepatic steatosis and steatohepatitis [4,95]. Although the pathogenic mechanisms underlying these relationships remain unclear, they are also thought to have their origins in adverse foetal epigenetic programming [93,94]. Similarly, while some studies have suggested that breastfeeding may be protective against the development of NASH in childhood, this likely depends greatly upon the physiological profile of the maternal source [4,96]. Others have also suggested that rapid weight gain, particularly in the first 3 months of post-natal life, rather than small birth size in and of itself, might increase the risk of NAFLD in childhood and later life, although further study is required to determine safe trends of neonatal weight gain [96].

### 3.3. Gender Differences and Puberty

In adults, numerous studies report that the prevalence of NAFLD, specifically simple steatosis, is twice as great in men as in women. While the exact reasons for these gender differences remain unclear, some have suggested that they might be explained by differences in fat distribution, serum lipid profile or a protective action of oestrogens and other hormonal differences between the sexes [14,97]. There are, however, no apparent gender differences in the risk of progression to NASH in adult or paediatric patients, although some studies have suggested that boys are more likely to develop a periportal paediatric pattern of NASH than girls [68].

However, in childhood and adolescence, gender differences appear to be more complex, with some studies supporting a higher risk in boys, similar to that in adults, while others do not. Instead, gender disparity with regards to NAFLD prevalence appears to increase with age and has been attributed to the physiological alterations that occur at the onset of puberty impacting the pathogenesis of this disease. Indeed, there is increasing evidence that associates rising levels of sex hormones during puberty with modification of diverse biological processes, including adipocyte development and function [4,24]. For example, animal studies have indicated that oestrogens reduce the severity of oxidative stress, impair hepatocellular mitochondrial function and inhibit hepatic stellate cell activation and fibrogenesis, which might significantly affect the development and progression of NAFLD by modifying the hepatic and systemic responses to hepatocellular injury [98,99,100]. Furthermore, the diminishing disparity in NAFLD prevalence between the genders, especially after middle age, has been widely noted, with some attributing it to hormonal changes that occur around menopause [101].

It has also been suggested that the rise in serum oestrogen levels in both boys and girls during puberty might also contribute to the reduced severity of NAFLD, particularly the more benign clinical course of simple steatosis, in adults. For example, in one study of 186 children with biopsy-proven NAFLD, after adjusting for confounders, patients at or beyond puberty were less likely to have high-grade steatosis, severe portal inflammation, borderline steatohepatitis (zone 1) or a high stage of fibrosis than patients who had not entered puberty [102]. There is also evidence to suggest that steatosis, inflammation and fibrosis are less severe during and after puberty among NAFLD patients [102].

### 3.4. Dysregulation of Hedgehog Signalling Pathway in NAFLD

Deregulation of the Hedgehog (Hh) Signalling Pathway, which morphologically orchestrates organogenesis during development, also appears to have a role in the pathogenesis and progression of NAFLD in adults and children [68]. While in the healthy adult this pathway is usually silent, it is reactivated when hepatic injury stimulates the production of Hh ligands, triggering the growth of various cell types involved in wound-healing, including resident hepatic immune cells, hepatic stellate cells and hepatic progenitor cells. While effective Hh signalling is necessary for injured mature livers to regenerate, prolongation or upregulation of this pathway’s activity has been linked to chronic inflammation, fibrosis and liver cancer [68].

Others have demonstrated that damaged or ballooned hepatocytes produce Hh ligands in adults with NASH, whose previous levels correlated with numbers of Hh-responsive cells within the liver and the severity of inflammation and fibrosis [103]. Whether or not similar mechanisms exist in children remains unclear but highly plausible, given that children generally harbour greater numbers of Hh-producing cells and Hh-responsive cells than adults and that these populations have been shown to expand even in response to relatively minor parenchymal injury, which may make them especially vulnerable to insults that stimulate liver damage and may even go some way towards explaining why simple steatosis has a much less benign course in children than in adults and why advanced fibrosis/cirrhosis can occur relatively rapidly [68].

Moreover, as hepatic development is not completed until adolescence, changes in the clinical presentation and course of NAFLD prior to and during adolescence, the latter being more in line with the adult pattern of disease, may reflect changes in the liver’s vulnerability to derangement of Hh pathway signalling [68]. It has even been suggested that age, gender and/or pubertal status may reciprocally influence Hh pathway activity in children, modulating the liver’s response to steatosis and hepatocyte injury and hence the histological features of paediatric NAFLD [12,68]. For example, in contrast to the adult liver, the periportal compartment of prepubescent male livers, where fibrosis characteristic of paediatric NAFLD is observed on histological analysis, exhibits high Hh pathway activity. Hh-mediated repair responses also appear to be more robust and readily engaged in prepubescent boys with NAFLD, which may explain why they display a much greater disease prevalence than girls [68].

Hh pathway activation also stimulates hepatic stellate cells to become myofibroblastic and function as the major collagen matrix-producing cells in response to liver injury. There is further evidence to suggest that, even once liver injury has dissipated and these cells revert to a quiescent state, they remain ”primed” to more readily reacquire their myofibroblastic and fibrogenic characteristics upon subsequent hepatic injury, which may further contribute to the aggressive pattern of paediatric NASH [104].

## 4. Making the Diagnosis

Paediatric NAFLD remains underdiagnosed due to a lack of recognition, under-appreciation of its associated complications or questions regarding the appropriateness of such a diagnosis in children by healthcare professionals. Far from being a process of exclusion, as it has often been described both clinically and in the literature, the diagnosis of NAFLD should be actively considered in all overweight or obese children >10 years old, particularly in the context of hypertension, evidence of hepatomegaly, acanthosis nigricans, insulin resistance and Type II diabetes mellitus [8,12,17,19,60].

**Figure 2:**
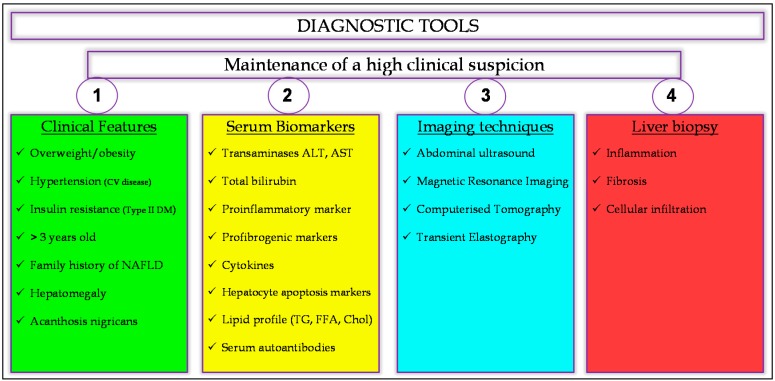
Diagnostic tools for children and adolescent NAFLD. Abbreviations: NAFLD: non-alcoholic fatty liver disease; DM: diabetes mellitus; ALT: alanine aminotransferase; AST: aspartate aminotransferase; TG: triglycerides; FFA: free fatty acids; Chol: cholesterol.

Differential diagnosis should first be based on the clinical features, then on blood tests, imaging techniques, and, finally, liver biopsy (Figure 2), which is currently considered the gold standard for the diagnosis of NAFLD [17], facilitating differentiation between simple steatosis and NASH, determining the presence and severity of hepatic fibrosis and providing prognostic information regarding the potential for disease progression [11,19,49]. Any evidence of hepatic steatosis in children <10 years old, with or without elevated liver function tests (LFTs), hepatomegaly or splenomegaly, is of particular concern and should be assessed comprehensively and expediently in order to exclude other aetiologies, including infectious hepatitis, autoimmune hepatitis, Wilson’s disease, haemochromatosis, α-1 antitrypsin deficiency and other monogenic causes of impaired fatty acid metabolism or lysosomal or peroxisomal storage. Despite being much less common in the paediatric population, Alcohol-induced Fatty Liver Disease must also be excluded and should not be discounted out of hand, even in young children [9,11,19,43,49].

Positive serum autoantibodies (anti-mitochondrial and anti-nuclear) are often present in paediatric NAFLD patients (~20%), even in the absence of autoimmune hepatitis, although their clinical significance remains unclear [12]. NAFLD is also often associated with abnormalities in iron metabolism, raising intra-hepatic free iron alongside mildly elevated serum ferritin and transferrin, in the absence of genetic haemochromatosis, seemingly mediated by pro-inflammatory adipokines. As such, liver biopsy is required in order to assess hepatic iron concentration and exclude significant hepatic injury and fibrosis, in patients with suspected NAFLD who demonstrate persistently elevated serum ferritin and increased transferrin saturation, especially in the context of homozygote or heterozygote C282Y mutations in the HFE gene [4,19,105]. Furthermore, due to its high prevalence, NAFLD can readily co-occur with other chronic liver diseases, worsening clinical outcomes that, otherwise, can be improved by concurrently treating the metabolic risk factors underlying NAFLD, such as obesity and insulin resistance [11,19].

Although possessing limited sensitivity, abdominal ultrasound and liver function tests remain the first choice in diagnosing NAFLD in children [11,19]. As such, while not recommended in the general paediatric population, biannual screening for elevated serum alanine aminotransferase (ALT) and aspartate aminotransferase (AST) should be undertaken in all obese patients above 10 years old, as well as those whose BMI falls between the 85th and 94th centiles and have associated metabolic risk factors. However, as a result of the pathophysiological and clinical differences between paediatric and adult NAFLD, diagnostic algorithms and risk prediction scores, such as the NAFLD activity score, which were developed for use in adults, are of limited utility in children and should not be relied upon [9,29]. Furthermore, radiological and histopathological findings should be interpreted with caution, as serum aminotransferase levels remain normal in the majority of paediatric cases, irrespective of disease severity and the often negligible levels of hepatic steatosis in advanced paediatric NASH rendering hepatic ultrasound insensitive. Even liver biopsy is not always reliable in paediatric NAFLD due to steatotic lesioning within the liver being less diffuse and characterised by much more subtle histopathological changes [17,19,106].

In the absence of definitive radiological and histopathological diagnostic tests, maintenance of a high clinical suspicion in both primary and specialist care settings and by all members of the multidisciplinary team remains the most potent of diagnostic tools, enabling early diagnosis and appropriate therapeutic intervention designed to stymie disease progression.

### 4.1. Alternative Classification System

The term ‘Non-alcoholic’, although originally intended to clearly differentiate the aetiology of this disease from Alcohol-Induced Fatty Liver Disease, is often extremely unhelpful and perpetuates the false assumption among healthcare professionals that paediatric NAFLD represents a diagnosis of exclusion. Furthermore, what constitutes the threshold of ”significant” alcohol consumption, particularly in paediatric cases, remains moot. Others have, therefore, suggested the adoption of ”Obesity-induced Liver Disease” as a replacement term but this could also prove misleading, given that, while obesity is the single greatest risk factor for this disease, NAFLD can develop in normo-weight children [27,35,107]. Such terminology is also likely to be the focus of significant social stigma, which is of particular concern in younger and more emotionally and psychologically vulnerable patients, potentially affecting their engagement with clinical services.

In light of the significant pathophysiological overlap between NAFLD and Alcohol-induced Fatty Liver Disease, it may be more helpful to think of “Fatty” Liver Disease or, less pejoratively, “Steatotic” Liver Disease (SLD) in terms of ”primary”, “secondary”, ”mixed” and “complex” aetiological subtypes. As such, “Primary” or “Type 1” SLD would encompass what is currently referred to as “Non-alcoholic Fatty Liver Disease”, which represents the phenotypic manifestation of underlying metabolic dysfunction in the absence of other causes of liver injury. “Secondary” or “Type 2” SLD would describe pathology resulting from a number of medical or surgical conditions or drug intake, including alcohol. In such cases where metabolic dysfunction and significant alcohol consumption coincide, the term “Mixed” or “Type 3” SLD could be used and, where Steatotic Liver Disease coincides with another form of chronic liver disease, such as autoimmune hepatitis, “Complex” or “Type 4” SLD. Thus, by appropriately reviewing the clinical nomenclature, we might better emphasise the importance of the diagnostic, pathophysiological, therapeutic and prognostic relationships between NAFLD and other chronic liver diseases in childhood and adolescence, as well as clearly directing intervention to improve clinical outcomes.

### 4.2. Serum Biomarkers for Liver Damage

Elevated levels of various circulating biomarkers have been described in patients with NAFLD, including AST and ALT, cytokeratin 18 (CK-18) fragments, apolipoprotein A1, total bilirubin, hyaluronic acid, C-reactive protein, fibroblast growth factor-21, interleukin 1 receptor antagonist, adiponectin, and TNF-α [83]. However, at present, there remains no readily available biomarker that reliably differentiates between simple steatosis and NASH.

Aminotransferases, AST and ALT, are the most commonly referenced serum biomarkers for liver damage in a wide variety of liver diseases, including NAFLD. They are easily obtained, low in cost and elevated levels have been associated across numerous studies with the presence and severity of NAFLD in adults [9,11,22,101]. Furthermore, in one multicentre study of 176 children, AST and GGT were predictive of both NAFLD and NASH but lacked the discriminatory power to accurately and reliably delineate cases of NASH from simple steatosis [108]. However, consensus as to what constitutes “normal” aminotransferase levels in children has yet to be established. Indeed, another study of 502 18–64 year olds with NAFLD demonstrated progressive decline of ALT levels with advancing age, while AST remained stable, suggesting that ALT elevation in childhood may be less diagnostically useful than in adult disease [101]. Most importantly, several studies have reported that up to two thirds of children with NASH did not display elevated serum ALT and AST levels, even in more advanced disease [109,110,111,112]. While normal AST and ALT levels do not exclude severe liver damage or fibrosis in paediatric NAFLD, when elevated they should inspire a high level of clinical suspicion, particularly in overweight or obese patients with a family history of NAFLD and, thus, may still be of significant use as a screening tool [9,74].

Elevated serum CK-18 fragments, markers of hepatocyte apoptosis, have demonstrated robust association with the incidence and severity of NASH in both adults and children [113]. Wieckowska *et al.* [114], for example, reported a strong positive correlation between CK-18 in plasma obtained from patients with suspected NAFLD at the time of liver biopsy and hepatic damage. Plasma CK-18 levels were also markedly increased in patients with NASH compared to those with simple steatosis, and were capable of accurately predicting NASH. These observations have been reproduced in subsequent studies, collectively suggesting CK18 levels to have a sensitivity of 78% and specificity of 87% for steatohepatitis in patients with NAFLD [115]. However, CK-18 would likely only be of use once the diagnosis of NAFLD had been made, as hepatocyte apoptosis is not unique to NAFLD. Furthermore, despite its significant clinical potential, CK-18 is not, at the present time, readily available and a standardised cut-off has yet to be established.

Total Bilirubin was also found by Puri *et al.* [116] to inversely correlate with the prevalence of NASH in children, which it is thought may reflect some anti-oxidative protective effect of bilirubin within the liver.

Finally, serum lipid profile, including total cholesterol, while potentially reflective of abnormal lipid metabolism that may contribute to NASH, has yet to be adequately investigated in paediatric liver disease. As such, its sensitivity, specificity and clinical utility remain unclear [53,117]. However, analysis of molecular lipid concentrations in blood samples taken from 679 adults found that those with NAFLD displayed increased triglycerols with low carbon number and double-bond content, while lysophosphotidylcholines and either phospholipids were diminished [118]. A serum lipid signature comprising these three molecular lipids had a sensitivity of 69.1% and a specificity of 73.8% in the subsequent validation series. Further investigation is required to validate these results in children, however.

### 4.3. Abdominal Ultrasound

Abdominal ultrasound is the most commonly used imaging modality for NAFLD, both clinically and in research [10,12]. It has been shown to be an effective means of identifying pure hepatic steatosis and mild NASH in children and has led to a great increase in findings of NAFLD in recent years. Its relatively low cost, wide availability and safety also make it an ideal screening tool [10,17]. In NAFLD, the liver is usually enlarged and appears echogenic, or “bright”, which indicates fatty accumulation within the parenchyma. However, it is unable to quantify the true extent of steatosis and its sensitivity diminishes significantly in cases where hepatic fat accumulation remains below 30%, in individuals who are severely obese (BMI > 40) and in severe NASH [9,44,119]. Ultrasound is unable to reliably differentiate between simple steatosis and steatohepatitis or exclude fibrosis. Accurately differentiating between focal steatosis or steatohepatitis and hepatic tumours or inflammatory vascular conditions is also challenging, given their close resemblance to one another on ultrasound and the potential for steatosis to obscure the imaging of other hepatic lesions [106,119]. However, while the focal manifestations of NAFLD may be characterised by poorly delineated margins and similar contrast enhancement with normal liver parenchyma, they do not exert a mass effect on the surrounding tissue and, at least in adults, favour certain topographical configurations, mainly occurring adjacent to the falciform ligament or ligamentum venosum, in the porta hepatis and gallbladder. Whether such distributions of focal fatty lesions hold true in paediatric NAFLD, however, remains to be established [11,119]. Furthermore, atypical focal fatty liver sparing can also mimic hepatic neoplasia, manifesting round or oval-shaped phenomena with clear margins. The diagnostic efficacy of abdominal ultrasound is also greatly dependent upon operator proficiency and lacks standard methods of interpretation for paediatric NAFLD, underscoring the importance of considering the wider clinical picture throughout the diagnostic process and selection of appropriate therapeutic intervention.

### 4.4. Magnetic Resonance Imaging

Unlike abdominal ultrasound, MRI exhibits high sensitivity and specificity for paediatric NAFLD and is able to differentiate, even in severely obese patients, between simple steatosis and NASH [17,20]. It is also able to quantify the distribution and severity of even mild steatosis and fibrosis throughout the entire liver and with moderate to strong correlation with histological grading in children and adults [28,120,121]. However, the relatively high cost of MRI, as well as the need for sedation in young children prohibits widespread use in clinical practice and, as such, it remains primarily a research tool. It is also, at present, unable to assess the extent of inflammation or cirrhosis in the liver parenchyma but rather identifies the consequences of chronic liver disease, such as hepatosplenomegaly and portal hypertension.

### 4.5. Other Imaging Techniques

While Computerised Tomography (CT) offers greater sensitivity than abdominal ultrasound in detecting the presence and extent of hepatic fat accumulation in NAFLD, the high radiation exposure it encumbers prohibits routine use in young children [20,121]. Furthermore, it also lacks the sensitivity required to detect mild steatosis and small changes in fat content over time.

Transient Elastography is able to detect hepatic fibrosis in paediatric NAFLD, using a technique similar to abdominal ultrasound to measure hepatic “stiffness” non-invasively. However, at present, it cannot reliably determine the extent or severity of hepatic fibrosis, particularly in its early stages, as both steatosis and inflammatory activity also marginally increase liver stiffness. This technique also suffers from diminished sensitivity and specificity in severely obese patients [122,123].

### 4.6. Liver Biopsy and Histopathology

Liver biopsy remains the gold standard for diagnosing NAFLD, differentiating between simple steatosis and NASH and determining the severity of liver damage, inflammation and fibrosis [19,29]. It also allows the clinician to rule out other causes of liver pathology, especially in cases of significant liver damage where abdominal ultrasound demonstrates reduced sensitivity and specificity. However, it is invasive and, as such, carries significant risks that render it unsuitable for use as a screening tool, particularly in children. It is also expensive and subject to sampling error, where subsequent histopathological analysis is unrepresentative of the liver as a whole. As such, even a normal liver biopsy cannot fully exclude NAFLD and should always be considered in context of the wider clinical picture.

The key decision pertains as to when biopsy is indicated and when it is not. In each case, the clinician must weigh the potential risks associated with biopsy against the likelihood that it will impact clinical management. Ideally, this would mean that we should only biopsy children who are at significant risk of NASH. However, our incomplete understanding of the natural history of this disease, at present, confounds any attempt to reliably stratify patients according to such risk, as the alteration of clinical outcomes based on the severity of histology at baseline remains unknown [124]. Nevertheless, current guidelines published by the American Association for the Study of Liver Diseases (AASLD) recommend that liver biopsy should only be undertaken in patients younger than 10 years old with a family history of severe NAFLD, the presence of hepatosplenomegaly at physical examination and abnormal laboratory results, encompassing transaminasaemia, insulin resistance, absence of autoantibodies and inconclusive results from biochemical tests for severe/progressive liver disease [19].

While children with NAFLD may exhibit the same morphological lesions as adults, these are often more subtle and can be absent altogether [44]. Hepatocyte ballooning, for example, which describes the enlargement of hepatocyte diameter by a factor of 1.5–2 and the main morphological feature of hepatocellular damage in adult NASH, is often not observed in paediatric cases. Similarly, the distinctive clarification and rarefication of hepatocyte cytoplasm and the inclusion therein of eosinophilic cytoskeletal peptide aggregates, referred to as Mallory Denk bodies, so characteristic of adult NASH, is relatively uncommon [11,12,19].

The distribution of fatty accumulation and fibrotic lesioning within the liver also differs between paediatric and adult disease. Adult NAFLD is characterised by microvacuolar periportal or panacinar hepatocellular steatosis, portal inflammation, portal fibrosis and perisinusoidal fibrosis. In contrast, paediatric NAFLD is characterised by macrovacuolar, azonal hepatocellular steatosis, portal inflammation and portal fibrosis [44,125].

Inflammation is characteristic of NASH across all age groups and comprises mixed inflammatory cells which infiltrate the hepatic parenchyma, including lymphocytes, histiocytes, Kupffer cells (KC) and granulocytes [63,64]. While, in adults, lobular inflammation is nearly universal and portal inflammation associated with more severe/advanced cases of NASH, portal inflammation is more typical of paediatric cases, providing further evidence that childhood disease follows a more severe course [125]. Furthermore, while isolated steatosis or steatosis with lobular inflammation without signs of hepatocellular injury are considered part of the wider spectrum of NAFLD in adults and insufficient evidence to suggest NASH, in children, where signs of hepatocellular injury are less obvious, this distinction is less clear. However, Schwimmer and others go on to describe both patterns of NAFLD in children, suggesting that factors other than age might determine the histological appearance of the disease [125]. Although the mechanistic underpinnings of this phenomenon remain unclear, Swiderska-Syn *et al.* [68] hypothesised that the Hedgehog pathway, which is involved in the fibro-ductal response, may effect such differences.

### 4.7. Non-Invasive Diagnostic Scoring Systems

The invasiveness, cost, morbidity and impracticality of liver biopsy in at-risk patients and especially in children has driven the development of non-invasive clinical risk prediction scores. However, many have yet to be validated in the paediatric population. Non-invasive hepatic fibrosis scores, AST/ALT, NFS and Fib-4 or AST/platelet ratio were developed for use in adults but have performed poorly in diagnosing significant fibrosis in children with NAFLD [29,126]. The paediatric NAFLD fibrosis index (PNFI) is calculated from the patient’s age, waist circumference and triglyceride levels and aims to predict liver fibrosis in children [126]. However, although it provides a good positive predictive value, its negative predictive value for ruling out fibrosis is sub-optimal. Several studies have suggested that the enhanced liver fibrosis (ELF) score, an algorithmic composite of serum markers of liver fibrosis, including hyaluronic acid, amino terminal propeptide of collagen type III and the tissue inhibitor of metalloproteinase, can be used to accurately predict fibrosis in children with NAFLD [126,127]. While the potential of these scores is great, their clinical utility remains, at present, unclear.

## 5. Management of Paediatric NAFLD

There is, currently, a lack of consensus as to how NAFLD in childhood and adolescence should be managed in clinical practice [65]. However, it is clear that effective therapeutic strategies should recognise that this is a multifactorial disease in which metabolic dysfunction is widespread, multifaceted, interdependent and is founded upon the interaction between numerous genetic and environmental forces. As such, therapeutic intervention should be adapted to each patient in context of their existing co-morbidities and how they might best be managed, including obesity, hyperlipidaemia, insulin resistance, Type II diabetes mellitus and cardiovascular disease. High clinical suspicion, enabling appropriate referral to paediatric gastroenterology, early diagnosis and intervention, has consistently been shown to be effective in improving overall quality of life for the patient, as well as reducing their long-term cardiovascular and hepatic morbidity and mortality [14,24,128].

**Figure 3:**
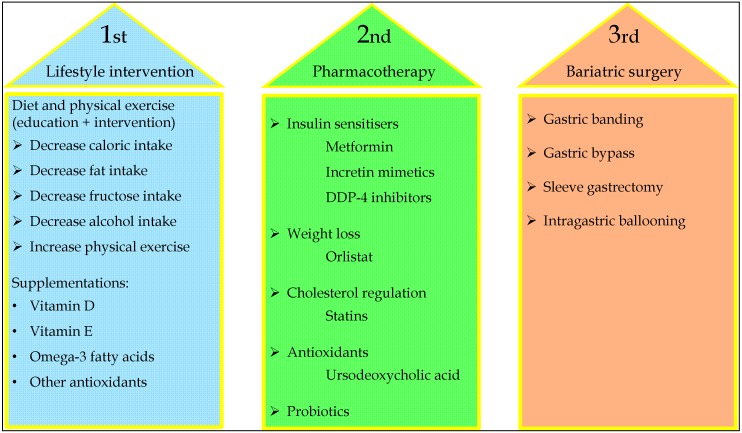
Management of paediatric NAFLD.

First-line interventions should focus on appropriately reducing central obesity and insulin resistance, primarily through dietary modification and increased physical exercise in order to effect therapeutic weight loss [129]. Depending on the extent of hepatic fibrosis, patients with NASH may also benefit from pharmacological therapies designed to slow or reverse disease progression [24]. Unlike in adults, where simple steatosis appears benign and, thus, pharmacological intervention is not recommended, in children the evidence suggests that it tends to follow a more aggressive course and, as such, pharmacological intervention, although not currently recommended, may be prudent before the transition to NASH occurs (Figure 3).

An approach that combines reducing visceral adiposity, insulin resistance and hyperinsulinemia with the prevention or reversal of hepatocellular damage appear to be the most successful rather than employing one or other of these strategies in isolation. The efficacy of any intervention should be assessed after a six-month period and, if ineffective, additional therapeutic options might then be considered, including pharmacological therapy or surgical intervention [24,65,128,130,131]. The development of comprehensive, evidence-based and internationally accepted clinical guidelines specifically for paediatric NAFLD will depend upon rectification of the current paucity of research and lack of robust epidemiological data. Nevertheless, they should emphasise the importance of the multidisciplinary team and the effective management of metabolic risk factors, as well as improving the interconnectedness of diverse health disciplines, especially during the transition from paediatric to adult clinical services and in those patients at the extreme end of the obesity spectrum, in whom non-surgical therapies for weight loss are currently non-existent.

Sleep shortage as a result of lifestyle, as well as major sleep disorders, such as sleep apnea and insomnia, have also been associated with NAFLD and may benefit from more active clinical consideration and therapeutic intervention. While the nature of these pathological relationships, remains the subject of much debate, various metabolic or endocrine effects in the context of obesity are thought to play a role [30].

### 5.1. Diet and Physical Exercise

Western diet, which is characterised by a hyper-caloric intake high in fats and simple sugars, precipitates a rapid increase in post-prandial plasma glucose and insulin levels, increasing hepatic *de novo*-lipogenesis, steatosis, insulin resistance, central obesity and the risk of NAFLD [21,132]. The Western Australian Pregnancy (Raine) Cohort Study (*n* = 995), for example, found that a Western dietary pattern at 14 years old was associated with an increased frequency of NAFLD at 17 years, independent of sex, dietary misreporting, family income, frequency of physical activity and sedentary behaviour [132]. As most paediatric patients with NAFLD are obese, addressing their obesity by means of dietary modifications, including reduction of caloric, fat and fast-release carbohydrate intake, as well as increasing physical exercise in order to effect weight loss should be considered the first-line of any effective interventional strategy. Indeed, current AASLD guidelines recommend limiting overall dietary fat intake to less than 5% of total caloric intake, while limiting trans-fats to <1% and saturated fats to <7% [74].

Numerous studies have shown that even a moderate reduction in weight, 5% in steatosis and 10% in NASH, has the potential to reduce hepatic steatosis, improve insulin sensitivity and significantly improve clinical outcomes in adults [14,19,44]. However, its effectiveness in patients with pre-existing NASH-induced hepatic fibrosis remains uncertain. Although few in number, paediatric studies seem to support these findings. One study in children with biopsy-proven NAFLD demonstrated that a reduction of 20% or more over 12 months precipitated significant improvement in serum ALT and steatosis in 68% of children [133]. Another study of 53 paediatric patients with NAFLD also reported significant reduction of steatosis, inflammation and hepatocyte ballooning on liver biopsy following similar lifestyle interventions [131].

Improvements may even be possible in a much shorter timeframe. Indeed, a recent Danish study of 117 obese children demonstrated marked improvement in their insulin sensitivity, liver fat accumulation and serum aminotransferase levels in two thirds of the cohort after only ten weeks of dietary intervention and one hour of moderate exercise daily [134]. Moreover, patients with NAFLD undertook less physical exercise than age and sex-matched controls and only 20%–33% of them met current recommendations for physical activity [135,136,137]. Physical activity correlates inversely with hepatic steatosis, independently of changes in body weight or dietary intake, increases insulin sensitivity and reduces central obesity, even in the absence of dietary alteration [11]. Furthermore, the extent of these changes appears, while apparent even in the short-term, to be proportional to the intensity and duration of lifestyle modification [135,138]. There is also evidence in adults to suggest that vigorous exercise is more beneficial than longer intervals of moderate exercise [139].

The minimum amount of weight loss necessary to improve clinical outcomes for patients with NASH, however, remains unclear. The current lack of specific clinical guidelines regarding which dietary modifications or physical exercise regimes would be most effective in inducing metabolic and histological improvement in children with NAFLD, beyond achieving weight loss in overweight children, perturbs a more systematic and evidence-based approach to the clinical management of this disease [19,24,65]. That said, any diet, whether alone or in conjunction with increased physical activity, that facilitates weight loss can effectively reduce hepatic steatosis, provided that the patient adheres to it. Early dietary intervention in childhood is especially important, given that dietary patterns formed in childhood tend to be retained into adulthood [132].

Lifestyle modification, however, can be difficult for younger patients to engage with and maintain long-term, particularly in the context of negative perceptions of dietary intervention and the prescription of physical exercise in children, among patients and their parents [9]. As such, lifestyle intervention should be tailored towards patients as individuals, taking account of the cultural and socioeconomic determinants of diet and exercise habits, as well as differences in patient perceptions of obesity and body image, particularly in adolescence, before setting clear and achievable goals derived by the patient and clinician in partnership. The adoption of similar lifestyle modifications by family members and, in some cases, behavioural therapy may aid compliance [15]. More effective and straightforward tools for monitoring day-to-day quality and quantity of dietary intake and physical activity in childhood, as well as greater efforts to educate and provide guidance for parents and their children regarding maintaining a healthy diet and the importance of physical activity are needed [140].

#### 5.1.1. Dietary Fructose

Besides the control of total caloric intake, the consumption of certain micronutrients, such as fructose, which is a constituent of sucrose, corn syrup, fruit juice, soft-drinks and various sweeteners, should also be reduced. Unlike glucose, fructose is metabolised exclusively in the liver and is preferentially shunted into the *de novo*-lipogenesis pathway via glyceraldehyde-3-phosphate, contributing to increased triglyceride synthesis and hepatic steatosis [141,142]. It has also been suggested that fructose may interact with nuclear transcription factors, such as sterol response element binding protein-1c, precipitating alterations in the expression of genes involved in liver glycolysis and lipogenesis [143]. It may also promote liver injury in NAFLD by causing bacterial overgrowth and increased intestinal permeability, precipitating endotoxemia and subsequent initiation of inflammation but this has yet to be proven [142,143].

In adults and in rodents, fructose has also been associated, particularly in the context of a high-fat diet, with a higher risk of NAFLD and increased liver fibrosis [21,44,142]. Moreover, the severity of hepatic steatosis and inflammation in rats fed fructose-enriched diets tends to be more severe than in controls [144]. Human studies also report greater fructose consumption in adult NAFLD patients and greater soft drink consumption and fasting serum triglyceride levels in children with NAFLD relative to controls [142]. Indeed, fructose consumption has dramatically increased in recent years and has also been associated with increased central obesity, dyslipidaemia and insulin resistance, all independent risk factors for NAFLD [27,145].

#### 5.1.2. Vitamin D

Vitamin D plasma levels have also been shown to inversely correlate with NASH and fibrosis in children and adolescents [146,147]. Furthermore, Vitamin D deficiency is more common in obese patients than those of normal weight and was shown to be associated with the incidence of NAFLD, liver steatosis, necroinflammation and fibrosis in adults [146,148].

Vitamin D receptors regulate the expression of numerous genes, some of which are involved in glucose and lipid metabolism, and are widely distributed throughout the liver [146,148]. In rats exposed to obesogenic diet, Vitamin D deficiency exacerbates NAFLD through the activation of Toll-like receptors and is associated with insulin resistance, hepatic inflammatory markers and oxidative stress [149].

Growing evidence also suggests that low serum Vitamin D is associated with insulin resistance and Type II diabetes and that appropriate Vitamin D supplementation can improve insulin sensitivity [150]. However, in the Western Australian Pregnancy (Raine) Cohort, others have reported the association of low Vitamin D levels with evidence of NAFLD on ultrasound at 17 years of age was independent of adiposity and insulin resistance [146]. As such, screening for Vitamin D deficiency in adolescents otherwise considered at high risk of NAFLD may be appropriate. Further clinical and experimental investigation of this phenomenon, as well as the benefits of dietary supplementation, is warranted [146].

#### 5.1.3. ω-3 Fatty Acids

Experimental models in animals and adults have shown that long chain ω-3 fatty acids, known important regulators of hepatic gene transcription, can decrease hepatic steatosis, improve insulin sensitivity and cardiovascular disease and decrease markers of inflammation [24,151,152].

Elsewhere, dietary depletion of polyunsaturated fats, such as ω-3, has been associated with the pathogenesis of NAFLD, while its progression has been associated with high circulating and hepatic levels of saturated fatty acids and industrial trans-fats. As such, limiting daily consumption of foods high in saturated fatty acids, while supplementing ω-3 intake may have a role in NAFLD treatment [9,153,154].

It is thought that the beneficial effects of ω-3 supplementation may be secondary to their known anti-inflammatory, antithrombotic, antiarrhythmic, hypolipidaemic and vasodilatory properties. There is evidence to suggest that they might also improve lipid profiles, lowering triglyceride serum levels, decreasing insulin resistance, hepatic steatosis and cytokine synthesis [153]. For example, dietary supplementation with docosahexaenoic acid (DHA), the major dietary long-chain polyunsaturated (ω-3) fatty acid, which exerts a potent anti-inflammatory effect through the G protein-coupled receptor 120 (GPR-120), has been associated with significant improvement in the histological parameters of NAFLD, including NAFLD activity score, hepatocyte ballooning and steatosis in children, after 18 months [154]. Interestingly, hepatic progenitor cell proliferation was also reduced in correlation with these same histological parameters, as were the numbers of inflammatory macrophages on biopsy, while GPR-120 expression in hepatocytes was markedly increased. As such, it was suggested that DHA might also modulate hepatic progenitor cell activation, hepatocyte survival and macrophage polarisation through interaction with GPR-120 and NF-κB repression [154]. Another study also described that, after 6 months of ω-3 supplementation, hepatic echogenicity and insulin sensitivity were significantly improved in children with NAFLD, although no change in serum ALT or BMI was observed [155]. More recently, another RCT reported the use of probiotics and ω-3 fatty acids showed encouraging early results, with improvement of serum liver enzymes but without validating liver histology [156]. AASLD guidelines currently state that it would be premature to recommend ω-3 fatty acids for the specific treatment of NAFLD or NASH but they may be considered first-line therapeutic agents to treat hypertriglyceridaemia in patients with NAFLD [19].

### 5.2. Alcohol

Heavy alcohol consumption is a risk factor for chronic liver disease and should be avoided in patients with simple steatosis and NASH [19]. There is even evidence to suggest that regular consumption of smaller quantities of alcohol (below 20 g/day) may be harmful [30]. However, there are no studies reporting the effect of ongoing alcohol consumption on disease severity or natural history of NAFLD or the risk of liver cancer in childhood and adolescence in the long-term.

### 5.3. Bariatric Surgery

Bariatric surgery has been shown to significantly improve weight and comorbid disease in patients with NAFLD. It encompasses a range of restrictive procedures, which promote satiety and delayed gastric emptying, including adjustable gastric banding and sleeve gastrectomy, malabsorptive procedures, including biliopancreatic diversion, and combinatorial procedures, such as Roux-en-Y gastric bypass [11,14,19,157].

At present, bariatric surgery is only recommended for severely obese adolescents with significant steatohepatitis in whom therapeutic lifestyle intervention has been unsuccessful [14,157]. In such patients, it has been shown to significantly reduce the extent and severity of hepatic injury, steatosis and systemic inflammation, as well as having broader metabolic benefits, improving insulin sensitivity, positively modifying levels of circulating adipokines and the intestinal microbiome, particularly in the case of malabsorptive procedures [14]. Furthermore, it has been suggested that malabsorptive procedures might also have additional effects on gut hormone profiles, reducing ghrelin, enhancing Glucagon-like peptide-1 (GLP1) secretion and facilitating early ileal exposure to nutrients, alongside reduced expression of peptide YY (PYY) and oxyntomodulin obesity-related genes and altered bile metabolism [14,158].

However, despite a large body of evidence suggesting histological improvement secondary to weight loss in adults, bariatric surgery in NASH patients of any age group remains controversial [11,12,14,19,159]. Indeed, a lack of randomised controlled studies, small sample sizes variable inclusion criteria, incomplete longitudinal follow-up and lack of clear identification of confounding factors, such as insulin resistance led the Cochrane meta-analysis to conclude that the impact of bariatric surgery on NASH in childhood and adolescence is unconvincing [160]. As such, current AASLD guidelines state that while bariatric surgery is not contraindicated in otherwise eligible obese patients, it is “not an established option for NASH treatment” [19].

Reports of *de novo* progression of NASH and even hepatic fibrosis and cirrhosis following bariatric surgery are also highly controversial [14]. Although some have sought to attribute this phenomenon to a state of “heightened metabolic stress”, in other surgical series, massive weight loss was shown to improve steatohepatitis and fibrosis. In this case, overall improvement was found to be dependent on the degree of insulin resistance, although long-term histological outcomes were not assessed [14,161].

Given the more aggressive nature of simple steatosis in paediatric disease, some have suggested that more earnest clinical intervention to reduce weight loss, including consideration of bariatric surgery, may be beneficial, even before the transition to steatohepatitis, in patients who are severely obese [12,28,159,160]. Further standardisation of eligibility criteria for surgery in the paediatric population, as well as studies on the safety and long-term efficacy of this approach, are warranted.

### 5.4. Pharmacological Intervention

Our understanding of the molecular pathogenesis of NAFLD remains limited and so current pharmacological intervention consists of strategies aimed at decreasing the incidence and severity of metabolic risk factors, such as obesity, insulin resistance, dyslipidaemia, as well as some drugs that target the major molecular pathways involved in the pathogenesis and progression of this disease of which we are aware, such as decreasing hepatic damage mediated by oxidative stress [67].

The aim of therapy is to forestall and, in some cases, reverse the progression of NAFLD to end-stage liver disease [11,162]. In particular, there remains a need for effective pharmacological therapies for children who do not adhere to or are unresponsive to lifestyle modification, in order to avoid severe organ damage [9,12].

Given the more aggressive clinical course of paediatric as opposed to adult NAFLD, targeted pharmacological intervention, although not presently recommended, may be prudent even before evidence of the transition to NASH is observed [11,14,19,28,68].

Collaboration between hepatologists and other relevant specialties, including endocrinology, paediatrics, dietetics, cardiology and primary care should be encouraged in order to optimise treatment, particularly in the current absence of clear clinical guidelines for pharmacological intervention in paediatric NAFLD.

#### 5.4.1. Insulin Sensitizers

Insulin resistance and Type II diabetes mellitus are strongly associated with the incidence, severity and progression of NAFLD in the paediatric population. As such, drugs that can improve insulin sensitivity have a key role in the prognostication and therapeutic management of this disease, potentially reversing even advanced liver damage and hepatic fibrosis, improving long-term clinical outcomes [24].

Metformin, an oral insulin-sensitising agent, lowers hepatic glucose production and promotes glucose uptake in the periphery and, when given in 500 mg doses twice daily for 24 weeks, has been shown to reduce hepatic steatosis on magnetic resonance spectroscopy and ALT levels in non-diabetic children with biopsy-proven NASH [163]. That said, while the Treatment of NAFLD in Children (TONIC) trial, in which a large non-diabetic paediatric cohort was used to compare metformin with Vitamin E therapy, found metformin to be no more effective than a placebo in achieving a sustained decrease in ALT levels, it did show significant improvement in hepatocyte ballooning [164]. Current AASLD guidelines do not recommend the prescription of metformin for NAFLD in non-diabetic paediatric patients [19]. Its effectiveness at doses higher than 500 mg twice daily, however, remains unknown. Moreover, specific guidelines for prescribing metformin in children and adolescents with NAFLD and Type II Diabetes are needed.

Pioglitazone, a Peroxisome-Proliferator Activated Receptor-γ (PPARg) agonist, increases insulin sensitivity and reduces hepatic fat content by promoting the redistribution of triacylglycerols from the liver and muscle to adipose tissue [19,24]. Therefore, while they have shown great promise in studies of adult NAFLD, their use often results in weight gain. Their safety and therapeutic efficacy in children, however, has yet to be determined and, indeed, there is a general reluctance to prescribe thiazolidinediones in paediatric patients, due to the potential side effects of long-term therapy, which include cardiotoxicity, fluid retention, osteoporosis and, as in adults, obesity [24].

Only glitazones have consistently shown some benefit in the treatment of patients with NASH in randomised-controlled trials [19,128]. Recent research suggests that pioglitazone can improve hepatic steatosis and inflammation, as well as reducing aminotransferase levels and histological evidence of hepatocyte injury in patients with biopsy-proven NASH [24,165,166]. However, the majority of patients in these trials were non-diabetic and, furthermore, the treatment had no apparent effect on the extent or severity of hepatic fibrosis.

Incretin mimetics and dipeptidyl peptidase-4 (DPP-4) inhibitors, which increase insulin secretion, decrease fatty acid oxidation and lipogenesis and improve hepatic glucose metabolism, may also have a role in NAFLD therapeutics [12,165,166,167]. DPP-4 is an enzyme implicated in the degradation of circulating GLP1, an incretin secreted in response to food intake that stimulates insulin secretion and inhibits glucagon release. Studies conducted in animals and adult humans have demonstrated the efficacy of GLP-1 receptor agonists, which were resistant to DPP-4 degradation, and DPP-4 inhibitors [12,167,168,169].

Suppressors of the renin-angiotensin system, such as losartan, reportedly improve insulin sensitivity and adipokine production/release and prevent hepatic stellate cell activation by exerting preventative effects on hepatic inflammation and fibrogenesis [12,168,170]. However, because of their contraindications, there is no available data on their therapeutic effects in children.

#### 5.4.2. Weight Loss Drugs

Orlistat, an enteric lipase inhibitor, is the only FDA approved therapy for weight loss in adolescents. It is moderately effective in achieving short-term weight loss but is limited in young patients due to adverse gastrointestinal side effects. However, despite several studies reporting improved ALT levels and hepatic steatosis in patients with NAFLD, others have failed to demonstrate histological improvement on biopsy. As such, their use in NAFLD remains controversial [171,172].

#### 5.4.3. Statins

Patients with simple steatosis and NASH are at increased risk of cardiovascular disease, with several studies having demonstrated this to be the most common cause of death in NAFLD. Effective therapeutic intervention in NAFLD, therefore, should encompass stratification of patients in terms of cardiovascular risk factors, including dyslipidaemia, and the appropriate clinical management thereof [11,19,27].

Despite general reluctance to prescribe statins to treat dyslipidaemia in patients with suspected or established chronic liver disease and the not uncommon occurrence of elevated aminotransferases in patients receiving statins, serious liver injury as a direct consequence of their use is rarely seen in clinical practice. Indeed, the risk of serious hepatic injury in patients with chronic liver disease, including NAFLD, does not appear to exceed that of patients without [173,174]. The evidence in children, however, remains less certain.

Several studies have thus far reported that statins can significantly improve liver biochemistries and cardiovascular outcomes in patients with elevated liver enzymes likely due to NAFLD. However, there remain no randomised-controlled trials with histological endpoints to support this either in simple steatosis or in NASH [11,174]. While current AASLD guidelines state that statins can be used to treat dyslipidaemia in adult patients with simple steatosis and NASH [19], their prescription in paediatric patients remains controversial.

### 5.5. Antioxidant Therapies

Oxidative stress is considered a key mechanism of hepatocellular injury and the progression of simple steatosis to NASH in children [88,175]. Given that, within hepatocytes, reactive oxygen species (ROS) are mostly generated in the mitochondria, some have suggested that, in hepatic steatosis, increased intracellular fatty acid levels may act as an overabundant substrate for mitochondrial malfunctioning, increasing ROS and, downstream, inflammatory cytokine and adipokine production, as well as, via their oxidation by peroxisomal acyl-CoA oxidases, the production of hydrogen peroxide, another reactive oxygen species [176,177].

Ordinarily, various enzymatic antioxidant mechanisms protect the liver from such oxidative injury, which in NAFLD, it seems, are simply overwhelmed. Therefore, the employ of antioxidant therapies would be expected to break this chain of lipid peroxidation and restore the endogenous antioxidant/oxidant equilibrium, halting the progression of NASH [148,178].

### 5.6. Vitamin E

Vitamin E therapy has been shown to reduce histological evidence of hepatic steatosis, inflammation and hepatocyte ballooning, as well as a reduction in aminotransferase levels in patients with NASH [148]. It has even been associated with the clinical resolution of steatohepatitis in adult NAFLD patients, although it does not appear to affect the extent or severity of hepatic fibrosis once it is established [179]. Studies in children have also reported improvement of liver function and glucose metabolism following a 12-month regime of Vitamin E (600 IU/day) and ascorbic acid (500 mg/day) in combination with dietary modification and physical exercise [133].

More recently, the NASH Clinical Research Network’s Treatment of NAFLD children (TONIC) trial, reported a modest benefit on hepatocyte ballooning following Vitamin E therapy in combination with similar lifestyle modifications in 8–17 year olds with biopsy proven NASH [164]. While aminotransferase levels were unaffected, statistically significant improvement of the NAS score and resolution of NASH with Vitamin E therapy was also observed over the following two years [164]. However, whether similar improvements can still be achieved in the absence of concurrent lifestyle modification remains controversial, as does the appropriate dosing of antioxidant therapies, including Vitamin E, in children. Indeed, there is some concern as to whether or not Vitamin E therapy increases all-cause mortality, as well as the risk of certain cancers, when administered in high doses [180].

While the most recent EASL guidelines advocate Vitamin E as a first-line pharmacotherapy in non-diabetic adults with biopsy proven NASH, the AASLD 2005 guidelines suggest that although Vitamin E also appears to be beneficial in non-diabetic children with NASH, confirmatory studies are needed before its use can be recommended in clinical practice. Furthermore, due to a similar lack of evidence, its use is not supported in diabetic patients with NASH, NAFLD without liver biopsy, NASH cirrhosis or cryptogenic cirrhosis at any age [11,19,148,162].

### 5.7. Ursodeoxycholic Acid

Ursodeoxycholic acid is one of the most widely used cytoprotective and antioxidant agents, able to protect hepatocytes from bile salt-mediated mitochondrial injury, as well as activating anti-apoptotic signalling pathways, fulfilling diverse immunomodulatory functions, in theory, stabilising cellular and organelle membranes in patients with NASH [24,181,182].

In children, a randomised controlled trial of ursodeoxycholic acid in combination with vitamin E therapy induced long-term improvements in liver function tests [183]. However, in another study of obese children with NAFLD, it was ineffective both alone and when combined with dietary intervention in decreasing serum ALT or the appearance of steatosis on ultrasound [184]. In another study in children, high doses of this acid induced a significant reduction in aminotransferase levels, although this was not the case with lower doses [185]. That said, its histological impact and therapeutic dose-threshold, as well as its effect on disease progression remains unclear. For example, in another study, two years of low-dose ursodeoxycholic acid in combination with vitamin E therapy was reported to improve biochemical and histological biomarkers [186]. Thus, the potential of ursodeoxycholic acid for reversing liver damage in paediatric NAFLD requires further attention.

### 5.8. Probiotic Therapy

Persistent cross-talk among the gut, the immune system and the liver appears to play an increasingly pivotal role in the pathogenesis and progression of NAFLD [63,64,154]. Emerging evidence suggests that specific nutrients are capable of increasing intestinal permeability to bacterial endotoxins, which, in turn, stimulate an immune-mediated inflammatory response from liver-resident cells, precipitating a profibrogenic phenotype. Several studies have also shown that the composition of the gut microbiome differs in NASH patients differs from that of obese patients without NASH and normoweight controls, specifically displaying a greater abundance of gram-negative bacteria [56,149].

Loguercio *et al.* [187] reported reduced hepatic injury and improved liver function tests following probiotic treatment in patients with various forms of chronic liver disease, including NAFLD. More recently, probiotic therapy in obese children with lactobacillus has been associated with significant improvement in serum aminotransferases and anti-peptidoglycan polysaccharide antibody levels, irrespective of BMI and visceral fat [188]. Further studies, have suggested that probiotics may reduce liver inflammation and improve gut epithelial barrier function. Probiotic therapy, therefore, represents a promising tool for the treatment of NAFLD in children by restoring the normal balance of gut microbiota [12,189].

Farnesoid X receptors (FXR), which are expressed in the bowel and liver, have also been implicated in the pathogenesis of NAFLD by mediating control of lipid and glucose homeostasis and bacterial flora growth and may, therefore, represent a novel therapeutic target [12,190].

## 6. Conclusions

Non-Alcoholic Fatty Liver Disease (NAFLD) is now the most common form of chronic liver disease, affecting 10%–20% of the general paediatric population and 50%–80% of those who are obese [27,35]. Within the next 10 years, it is expected to become the leading cause of liver pathology, liver failure and indication for liver transplantation in childhood and adolescence in the Western world [19,29,49,117]. Despite this, “paediatric” NAFLD remains under-studied, under-recognised and, potentially, undermanaged. Important gaps remain in our overall approach to screening, diagnosis, management and follow-up, particularly during the transition between paediatric and adult clinical services and in those patients at the extreme end of the obesity spectrum, in whom non-surgical therapies for weight loss are currently non-existent [9,11,44].

The importance of raising clinical and public awareness of NAFLD in childhood and adolescence, as well as addressing widespread misconceptions regarding its prevalence, natural history and prognosis among healthcare professionals at all stages of their training and in light of emerging evidence, cannot be overstated. The strong association between paediatric NAFLD and metabolic risk factors, including insulin resistance, dyslipidaemia, cardiovascular disease and, most significantly, obesity, highlights the need for greater interconnectedness and collaboration between diverse clinical specialties and the potential for significantly improving patient outcomes through targeted dietary modification, reduction of caloric intake, increased physical exercise and, where appropriate, pharmacological therapy [9,21,67,131].

The current paucity of research in paediatric NAFLD has perpetuated a limited understanding of its pathophysiology and hampered the selection and development of more effective therapeutic interventions since this disease was first described in children in the mid-1970s. More accurate epidemiological data derived from longitudinal and larger cohort studies will be needed in order to determine the true prevalence of NAFLD in childhood and adolescence and allow the development of more accurate risk prediction scores to augment clinical screening and surveillance, as well as comprehensive clinical guidelines specifically for the diagnosis and management of paediatric disease, which are currently lacking.

By appropriately reviewing the nomenclature, we might better emphasise the importance of the clinicopathological relationships between NAFLD and other chronic liver diseases in childhood and adolescence.

In the absence of definitive radiological and histopathological diagnostic tests, maintenance of a high clinical suspicion in both primary and specialist care settings and by all members of the multidisciplinary team remains the most potent of diagnostic tools, enabling early diagnosis and appropriate therapeutic intervention.

## Abbreviations

LD	Linear dichroism
AASLD	American Association for the Study of Liver Diseases
ALT	Alanine Aminotransferase
AMPK	AMP-activated Protein Kinase
APOC3	Adipoprotein C3
AST	Aspartate Aminotransferase
BMI	Body Mass Index
CB2	Cannabinoid Receptor 2
CK-18	Cytokeratin 18
CT	Computerized Tomography
CYP2E1	Cytochrome P450 family 2 subfamily E member 1
DGAT2	Diacylglycerol O-Acyltransferase 2
DHA	Docosahexaenoic Acid
DPP-4	Incretin Mimetics and Dipeptidyl Peptidase-4
EASL	European Association for the Study of the Liver
ELF	Enhanced Liver Fibrosis
FTO	Fat Mass and Obesity associated
FXR	Farnesoid X Receptor
GCKR	Glucokinase Regulatory Protein
GGT	γ-glutamyl Transferase
GLP1	Glucagon-Like Peptide-1
GRP-120	G Protein-coupled Receptor 120
GWAS	Genome-Wide Association Study
HDL	High-Density Lipoprotein
HPC	Hepatic Progenitor Cells
HSC	Hepatic Stellate Cells
IL-6	Interleukin 6
IR	Insulin Resistance
IRS-1	Insulin Receptor Substrate-1
IUGR	Intrauterine Growth Restriction
KC	Kupffer Cells
KLF-6	Kruppel-Like Factor 6
LDL	Low Density Lipoprotein
LFTs	Liver Function Tests
LPIN1	Lipin 1
LYPLAL1	Lysophospholipase-Like 1
MC4R	Melanocortin 4 Receptor
NAFLD	Non-Alcoholic Fatty Liver Disease
NASH	Non-Alcoholic Steatohepatitis
NCAN	Neurocan
NKT	Natural Killer T lymphocytes
PNFI	Pediatric NAFLD Fibrosis Index
PNPLA3	Adiponutrin/Patatin-like Phospholipase Domain-containing 3
PPARg	Peroxisome-Proliferator Activated Receptor-γ
PPP1R3B	Protein Phosphatase 1 Regulatory Subunit 3b
PYY	Peptide YY
ROS	Reactive Oxygen Species
SLD	Steatotic Liver Disease
SOD2	Manganese-dependent Superoxide Dismutase
SREBP1c	Sterol Regulatory Binding Element
TNF-α	Tumor Necrosis Factor α
TONIC	Treatment of NAFLD in Children
